# Comparison of tibial cortex transverse transport and free anterolateral thigh perforator flap in the treatment of severe diabetic foot ulcers: a retrospective study

**DOI:** 10.3389/fsurg.2026.1742244

**Published:** 2026-03-09

**Authors:** Shunan Dong, Jiyong Jiang, Sijie Yang, Qikai Hua

**Affiliations:** 1 Department of Joint Surgery, The First Affiliated Hospital of Guangxi Medical University, Nanning, China; 2 Department of Upper Extremity Trauma, Reconstruction and Plastic Surgery, Beijing Jishuitan Hospital Guizhou Hospital, Guiyang, China; 3 Department of Orthopedics, Guizhou Provincial People’s Hospital, Guizhou, China; 4 Department of Orthopedics, The People’s Hospital of Guangxi Zhuang Autonomous Region, Nanning, China

**Keywords:** anterolateral thigh perforator flap, diabetic foot, limb reconstruction, tibial cortex transverse transport, ulcer

## Abstract

**Introduction:**

Free anterolateral thigh perforator flap (ALTPF) reconstruction is a conventional approach for the treatment of diabetic foot ulcers (DFU), whereas tibial cortex transverse transport (TTT) represents an emerging alternative. However, direct comparative studies evaluating their therapeutic efficacy remain limited.

**Methods:**

A retrospective analysis was performed on patients with DFU treated at the First Affiliated Hospital of Guangxi Medical University between January 2016 and December 2022. All patients underwent either TTT or ALTPF reconstruction. Treatment and follow-up data were obtained from the hospital's Computer Information Center and the outpatient follow-up system. Patient demographics and wound-related information were collected. Ulcer healing status was assessed using wound photographs and follow-up records, with healing time, recurrence, and amputation events documented. Foot sensory function was evaluated using the Semmes–Weinstein monofilament test (SWMT) and nerve conduction velocity measurements, while postoperative foot function was assessed according to the Maryland Foot Score.

**Results:**

A total of 174 patients with DFU were included in this study, of whom 88 underwent TTT and 86 received ALTPF reconstruction. The TTT group had significantly shorter operative time, less intraoperative blood loss, and a lower transfusion rate than the ALTPF group (*P* < 0.05). The ulcer healing rate was higher in the TTT group (98% vs. 88%, *P* = 0.015), whereas the recurrence and major amputation rates were significantly lower (both *P* < 0.05). The TTT group also showed a higher rate of positive SWMT and faster nerve conduction velocity compared with the ALTPF group (*P* < 0.05), along with better Maryland Foot Scores. Two cases of pin-tract infection occurred in the TTT group, while flap necrosis developed in nine cases in the ALTPF group.

**Conclusions:**

TTT demonstrated superior therapeutic efficacy to ALTPF in the management of severe DFU. TTT offered advantages including shorter operative time, reduced blood loss, lower transfusion and complication rates, higher healing rate, and better functional recovery. However, further randomized controlled trials are warranted to validate these findings.

## Introduction

With population ageing and changes in lifestyle, the prevalence of diabetes continues to rise and has become a major global public health concern. According to the International Diabetes Federation (IDF), approximately 537 million people worldwide are living with diabetes, and this number is projected to increase to 738 million by 2045 (1). Diabetic foot ulcer (DFU) is among the most serious complications of diabetes, characterized by lower-limb infection, ulceration, and deep tissue destruction, with or without peripheral arterial disease or neuropathy (2, 3). Severe DFU, generally defined as Wagner grade 3 or higher, not only profoundly impairs patients' quality of life but may also lead to amputation or even death (4). Conventional approaches for the management of DFU, including pharmacological therapy, physical therapy, and the use of biological agents, often show limited efficacy in patients with severe DFU (5, 6).

With the rapid advancement of microsurgical techniques, the anterolateral thigh perforator flap (ALTPF) has been widely used over the past decades for the reconstruction of various defects in the foot and ankle. ALTPF offers several advantages, including minimal donor-site morbidity, reliable vascularity, a large harvestable area, and favorable postoperative functional recovery (7–9). Previous studies have also demonstrated that appropriate application of ALTPF for DFU reconstruction achieves high flap survival rates, excellent limb salvage, and satisfactory restoration of foot function (10, 11).

In recent years, tibial cortex transverse transport (TTT) has emerged as an innovative surgical technique that has garnered increasing attention in the academic community (12–14). A growing body of evidence suggests that TTT can significantly enhance blood perfusion in the foot, promote microcirculatory reconstruction, and thereby accelerate the healing of DFU, effectively reducing or preventing amputation. Moreover, TTT is a relatively simple and minimally invasive procedure, which has encouraged clinicians to gain a deeper understanding and broader application of this novel technique.

Although both techniques have distinct advantages, there remains a lack of systematic evidence regarding which approach is more suitable and effective for the reconstruction of DFU. In this study, we retrospectively analyzed data from patients with severe DFU treated at the First Affiliated Hospital of Guangxi Medical University between January 2016 and December 2022. The study aimed to compare the strengths and limitations of these two techniques in the management of severe DFU and to provide new insights into the treatment of this challenging condition.

## Methods

### Study design and participant data collection

Clinical data and imaging records for this study were obtained from the Computer Information Center and the clinical follow-up system of the First Affiliated Hospital of Guangxi Medical University.

**Figure 1:**
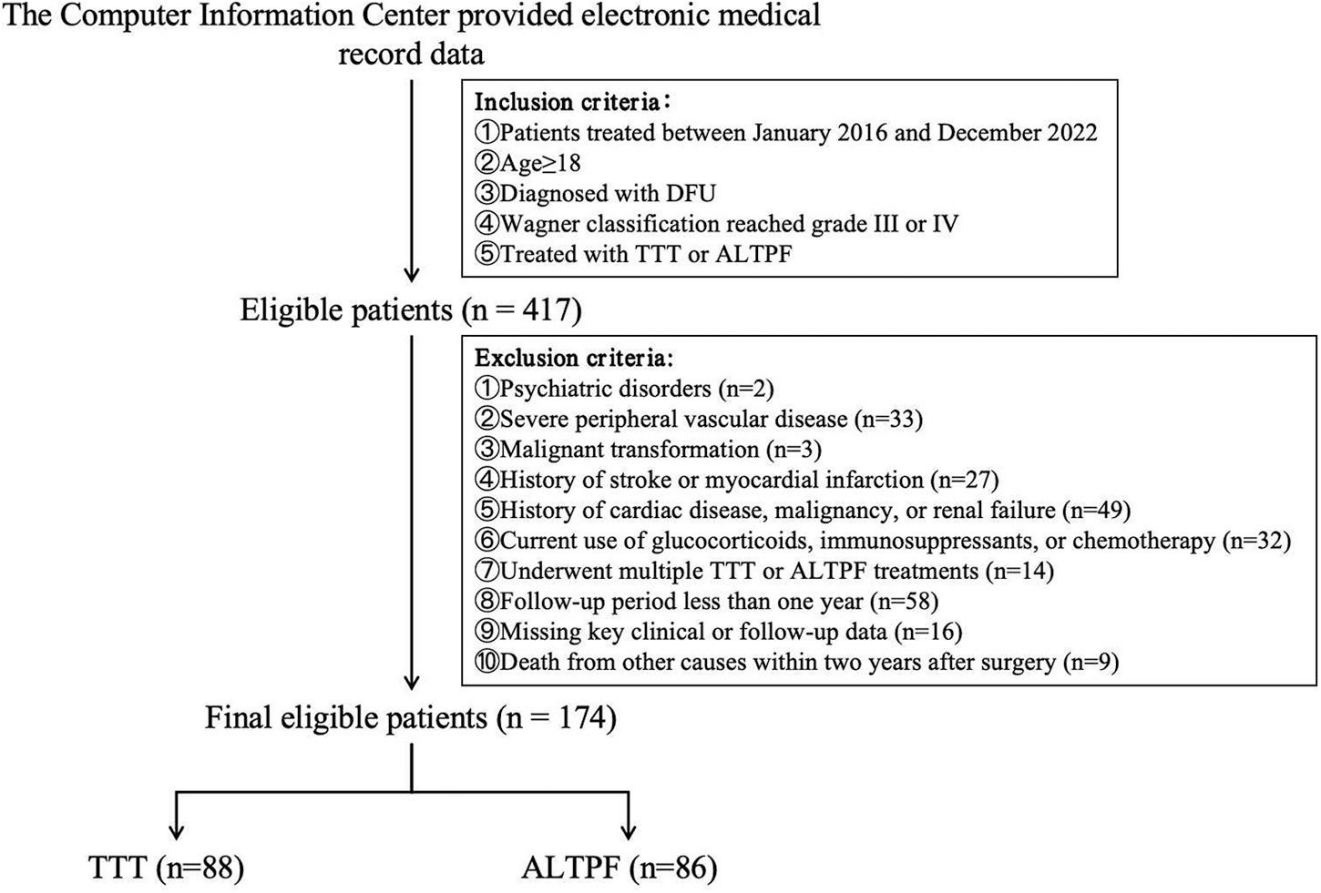
Clinical data collection flowchart.

As illustrated in Figure 1, this study retrospectively enrolled patients with DFU treated at the First Affiliated Hospital of Guangxi Medical University between January 2016 and December 2022. Briefly, patients aged 18 years or older who met the diagnostic criteria for DFU and had a Wagner classification of grade 3 or 4 were included (4). All patients received standard medical management, including debridement and wound care, in addition to either TTT or ALTPF reconstruction.

Patients were excluded if they were diagnosed with or suspected of having psychiatric disorders; had severe peripheral vascular disease (e.g., ≥80% popliteal artery stenosis not amenable to revascularization); had suspected or confirmed malignant transformation of the ulcer; had experienced stroke or myocardial infarction within the past six months; or had a history of cardiac disease, malignancy, or renal failure. Those receiving glucocorticoids, immunosuppressants, or chemotherapy, as well as patients who died from unrelated causes within two years after surgery, were also excluded. Additionally, patients who underwent multiple TTT or ALTPF procedures, had a follow-up duration of less than one year, or had incomplete key clinical or follow-up data were excluded.

The collected information primarily included patients' demographic characteristics, treatment details, and follow-up data. The main variables were sex, age, body mass index (BMI), smoking history, duration of diabetes, ulcer area, Wagner classification, Maryland Foot Function Score (MFS), ankle–brachial index (ABI), history of peripheral arterial disease (PAD), peripheral neuropathy, chronic kidney disease (CKD), osteomyelitis, and previous treatment history.

At both preoperative and three-month postoperative time points, patients' data on the MFS, Semmes–Weinstein Monofilament Test (SWMT), ABI, and nerve conduction velocity (NCV) were recorded.

BMI was calculated as weight (kg) divided by height squared (m²). The duration of diabetes was defined as the time interval from the initial diagnosis of diabetes to the date of surgical intervention (TTT or ALTPF). Ulcer area was determined based on clinical records and wound photographs. The Wagner classification was assessed according to the Wagner–Meggitt grading system for DFU (15).

The MFS comprehensively evaluates pain, gait, stability, appearance, brace dependence, range of motion, and walking distance to assess lower-limb functional recovery and ambulatory ability. The total score is 100, with higher scores indicating better foot function. To minimize subjective bias, the scoring was performed independently by two trained assessors, and the average of their evaluations was used as the final score.

PAD was assessed by lower-limb color Doppler ultrasonography or computed tomographic angiography (CTA). A reduction of more than 50% in the luminal diameter of the affected artery or the presence of a monophasic waveform on Doppler imaging was considered indicative of PAD (16).

Peripheral neuropathy was evaluated using the SWMT. It was performed to evaluate protective sensation of the foot. A 10-g monofilament was applied perpendicularly with sufficient pressure to bend the filament for approximately 1 s at specific anatomical sites on the plantar surface, including the pulp of the hallux and the heads of the first, third, and fifth metatarsals. Each site was tested twice, and patients were instructed to close their eyes and respond verbally when they felt the touch. Loss of sensation at ≥4 out of 10 tested sites (or ≥1 out of 4 in the simplified version), or absence of sensation at key sites such as the hallux or first metatarsal head, was defined as a positive result, indicating peripheral sensory neuropathy. A negative result indicated intact protective sensation (17).

The ABI was used to evaluate arterial perfusion of the lower limbs. After resting in the supine position for at least 10 min, systolic blood pressure was measured at both brachial arteries and at the ankle arteries of both lower limbs (including the posterior tibial and dorsalis pedis arteries). The ABI was calculated as follows:

ABI = ankle systolic pressure/brachial systolic pressure.

For each leg, the higher ankle systolic pressure and the higher brachial systolic pressure were used for calculation. An ABI value < 0.9 was considered indicative of peripheral arterial disease, 0.9–1.3 was defined as normal, and values >1.3 suggested arterial stiffness or noncompressible arteries (18).

CKD was identified based on the diagnoses documented in the medical records. For open wounds, a probe-to-bone test was performed and supplemented with radiographic examination to assess the presence of osteomyelitis (19).

The primary outcome measures included operative time, intraoperative blood loss, need for blood transfusion, ulcer healing, limb salvage, ulcer recurrence, recovery of foot function, recovery of foot sensation, and postoperative complications. Detailed information on operative time, blood loss, and transfusion status was obtained from surgical records.

Ulcer healing was defined as complete epithelialization of the wound without exudation, infection, or scabbing, maintained for at least two consecutive weeks without the need for further intervention or dressing coverage. The healing status was confirmed by two independent assessors through direct visual inspection and wound photographs. Because the ALTPF involves flap coverage, the ulcer healing time could not be reliably determined from photographs or clinical records in this group and was therefore considered not applicable. In the ALTPF group, the transferred flaps demonstrated sustained satisfactory perfusion throughout the first 14 postoperative days, as evidenced by a normal flap colour, capillary refill time, temperature, and turgor, with no dusky discolouration or blister formation; these findings were taken to indicate flap survival. This was also considered indicative of ulcer healing.

Limb salvage was defined as the absence of amputation during the follow-up period. Ulcer recurrence was defined as the occurrence of a new skin breakdown—deeper than the epidermis and requiring medical intervention or dressing coverage—either at the previously healed site or elsewhere on the same foot. Recurrence was recorded only after complete wound healing and was confirmed by two independent assessors based on follow-up records or wound photographs (20).

Foot function was evaluated using the MFS. Foot sensation was assessed with the SWMT and NCV measurements. NCV served as an objective indicator of peripheral nerve function in the lower limbs. All tests were performed with the patient in a resting state using an electromyography system (Nicolet system) to record both motor and sensory nerve conduction parameters.

### Ethical approval and informed consent

This retrospective study was approved by the Ethics Committee of the First Affiliated Hospital of Guangxi Medical University. All data were obtained from the hospital's electronic medical record system, extracted by the Information Center, and anonymized before analysis. As the study involved no direct contact with or intervention in patients, the requirement for informed consent was waived by the Ethics Committee. The study was conducted in accordance with institutional and national research ethics standards and adhered to the principles of the Declaration of Helsinki.

### Surgical procedure and postoperative management

#### TTT

**Figure 2:**
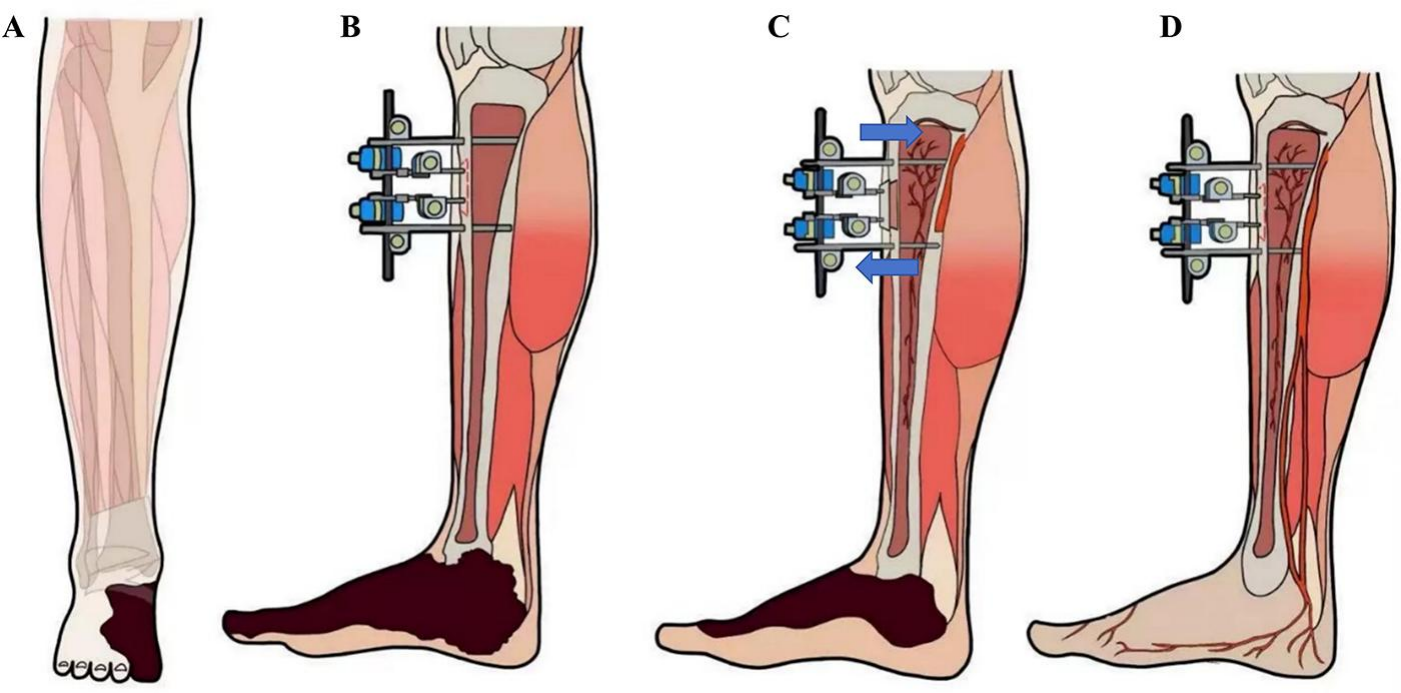
Key surgical steps of tibial cortex transverse transport (TTT) for the treatment of diabetic foot ulcer (DFU). **(A,B)** The bone fragment was created at the relatively flat, medial aspect of the proximal tibia (indicated in red). The inner two fixation pins secured the bone fragment, while the outer two pins stabilized the tibia; **(C)** The position of the bone fragment was adjusted vertically by regulating the movement of the inner fixation pins; **(D)** During distraction, neovascularization occurred not only around the bone fragment but also in the distant wound area, thereby promoting wound healing.

As described in our previous studies (12). In brief, patients in the TTT group underwent surgery under spinal or femoral nerve block anesthesia in the supine position without the use of a tourniquet. Two longitudinal incisions (approximately 1 cm each) were made over the anteromedial aspect of the proximal tibia. The skin and subcutaneous tissue were incised to expose the periosteum, and surrounding soft tissues were carefully dissected. A bone fragment measuring 5 cm in length and 1.5 cm in width was designed. Using a micro-oscillating saw, corticotomy was performed along the four margins of the fragment, penetrating only one cortical side. A 3 mm Kirschner wire was inserted into the fragment, followed by two 5 mm Schanz pins placed on either side, traversing both cortices. An external distraction device was then assembled, and intraoperative fluoroscopy was used to confirm the positions of the fixation pins and the integrity of the fragment (Figure 2).

Postoperatively, aggressive wound debridement was performed according to the Infectious Diseases Society of America (IDSA) guidelines. Wound photographs were taken by trained personnel who were not involved in the surgical procedure, and ulcer area was measured accordingly. Patients with wound infection received empirical antibiotic therapy guided by culture and sensitivity testing. Infected bone tissue was debrided intraoperatively, and patients were treated with a 6-week course of targeted antibiotic therapy (21).

Dressing changes and pin site care were performed daily. Postoperative radiographs were routinely obtained to confirm the corticotomy site and the position of the fixation pins. After a 3-day latency period, transverse tibial corticotomy distraction was initiated at a rate of 0.25 mm every 6 h. Patients were instructed to continue distraction at home after discharge: 14 days of medial distraction followed by 14 days of lateral distraction to return the bone segment to its original position. Follow-up radiographs were obtained during the second and fourth weeks of distraction to verify the position of the cortical segment.

All patients received standard daily wound care and off-loading plaster immobilization. Because the external fixation device provided stable fixation, patients were allowed partial weight-bearing with the assistance of a walker during the early postoperative period. After four weeks of distraction therapy, the external fixation device was removed in the outpatient clinic.

#### ALTPF

All patients in the ALTPF group underwent surgery under general anesthesia in the supine position. The procedure began with thorough debridement of the foot ulcer, followed by proximal exploration and marking of the recipient arteries and veins. A template was prepared according to the size and shape of the wound. The flap was then designed with the perforator of the descending branch of the lateral circumflex femoral artery, as identified preoperatively by color Doppler ultrasonography (CDU), serving as the central axis. The flap was planned to fully cover the defect with an adequate margin to allow for intraoperative adjustment. For large defects where primary closure of the donor site was not feasible, a lobed flap was designed to “convert width into length.”

**Figure 3:**
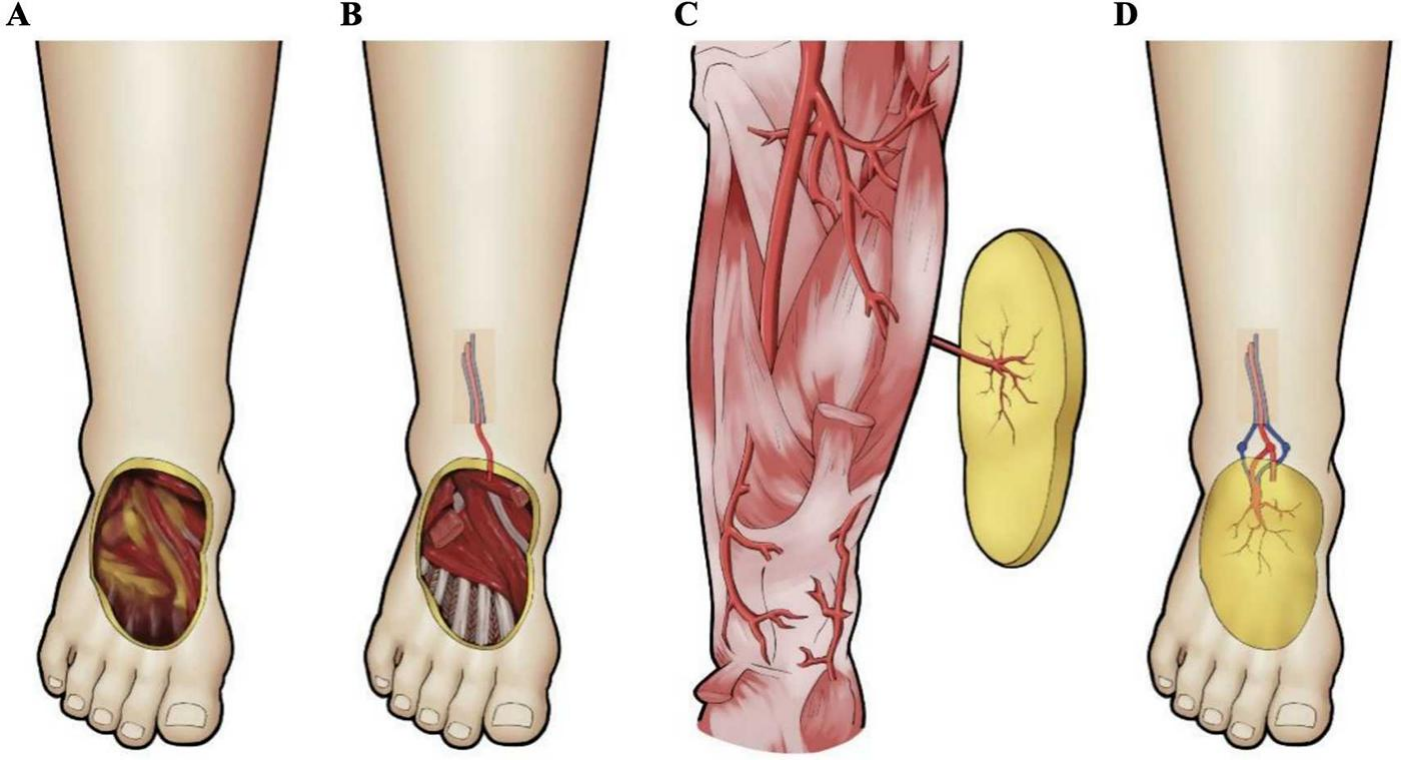
Key surgical steps of anterolateral thigh perforator flap (ALTPF) transplantation for the treatment of DFU. **(A,B)** Thorough debridement of the foot wound was performed to expose the recipient arteries and veins for anastomosis; **(C)** A skin flap containing vascular pedicles was designed and harvested according to the wound size; **(D)** The flap was transferred to the recipient site of the foot, with end-to-side anastomosis between the pedicle artery and the recipient artery, and end-to-end anastomosis between the pedicle vein and the recipient vein.

The skin and subcutaneous tissue were incised along the lateral margin of the flap, and the superficial fascia was dissected to expose the fascia lata. Dissection was carried out between the rectus femoris and vastus lateralis muscles toward the marked point, using a retrograde approach to trace the perforator to its source vessel. After complete flap elevation with a vascular pedicle of sufficient length, the proximal descending branch of the lateral circumflex femoral artery was ligated and divided. Hemostasis was achieved, and the donor site was closed in layers. The harvested flap was transferred to the DFU wound bed, adjusted for position, sutured intermittently, and the vessels were anastomosed. All flaps were routinely harvested with the lateral femoral cutaneous nerve of the thigh and coapted to the recipient-site cutaneous nerve to facilitate sensory reinnervation. The flap artery was anastomosed end-to-side to the anterior or posterior tibial artery, and the flap vein was anastomosed end-to-end to the accompanying vein (Figure 3).

After confirming adequate flap perfusion, meticulous hemostasis was performed again, the wound was closed, and a drainage tube was placed. The area was loosely covered with sterile dressings, and the affected limb was immobilized with a plaster splint. Following free flap reconstruction, patients were required to maintain absolute bed rest for seven days. Both groups received identical standards of wound care and off-loading management postoperatively (22).

Low–molecular-weight heparin was routinely administered postoperatively to prevent thrombosis. The flap was kept warm using an infrared heating lamp, and its condition was closely monitored, including capillary refill time, temperature, and the presence or absence of blister formation. Dressings were changed promptly when necessary. If vascular compromise of the flap was suspected, the underlying cause was identified immediately and managed accordingly, with surgical exploration performed when necessary. Patients were advised to avoid both active and passive smoking and to limit excessive movement of the affected limb. The rubber drainage strip was removed on postoperative day 2, and sutures were removed on day 14.

### Postoperative follow-up

During the first three postoperative months, patients were followed up weekly in the outpatient clinic for wound inspection, evaluation, and dressing changes. In the TTT group, gradual weight-bearing ambulation was permitted four weeks after removal of the external fixation device. In the ALTPF group, progressive weight-bearing was allowed after suture removal. After the initial three months, outpatient follow-up visits were scheduled every two months, with a minimum follow-up duration of one year.

### Statistical analysis

Clinical data were tested for normality using the Shapiro–Wilk test. For normally distributed variables, comparisons between groups were performed using the *t*-test; for non-normally distributed variables, the Mann–Whitney *U*-test was applied. Categorical variables were compared using the chi-square test or Fisher's exact test (when the expected frequency in any cell of the contingency table was <5). Continuous variables were expressed as mean ± standard deviation (mean ± SD), and categorical variables were presented as counts and percentages. Statistical significance was defined as *α* = 0.05. All statistical analyses were performed using SPSS version 25.0 (IBM Corp., Chicago, IL, USA).

## Results

As showed in Tables 1, 2, after screening, a total of 174 patients were included in this study, with 88 undergoing TTT and 86 receiving ALTPF. Intraoperatively, the TTT group had a significantly shorter operative time than the ALTPF group (59.31 ± 18.35 min vs. 274.26 ± 43.94 min, *p* < 0.05). The TTT group also demonstrated less intraoperative blood loss (12.11 ± 4.66 mL vs. 355.76 ± 144.22 mL, *p* < 0.05) and a markedly lower transfusion rate [3.41% (3/88) vs. 43.02% (37/86), *p* < 0.05].

**Table 1:** Comparison of clinical baseline characteristics between the two groups.

Variable	TTT (*n* = 88)	ALTPF (*n* = 86)	*p*-value
Age (years)	40.93 ± 15.53	45.15 ± 16.48	0.084
Sex [*n* (%)]			0.347
Male	47 (53.41)	52 (60.47)	
Female	41 (46.59)	34 (39.53)	
BMI (kg/m^2^）	24.15 ± 3.69	25.23 ± 4.17	0.073
Duration of diabetes (years)	17.45 ± 7.37	18.79 ± 7.77	0.246
Wound area (cm^2^)	59.91 ± 20.60	56.05 ± 18.74	0.198
Wagner class, *n* (%)			0.985
3	39 (44.32)	37 (43.02)	
4	49 (55.68)	49 (56.98)	
MFS	52.77 ± 9.85	52.69 ± 8.50	0.950
PAD [*n* (%)]	59 (67.05)	66 (76.74)	0.915
Peripheral neuropathy [n (%)]	64 (72.73)	63 (73.26)	0.786
NCV (m/s)	21.77 ± 5.82	20.05 ± 7.76	0.0596
ABI	0.85 ± 0.21	0.82 ± 0.23	0.364
CKD [*n* (%)]	27 (30.68)	31 (36.05)	0.453
Osteomyelitis [*n* (%)]	46 (52.27)	53 (61.63)	0.213
Smoking [*n* (%)]	31 (35.23)	40 (45.51)	0.130
Previous treatment [n (%)]			
Debridement	26 (29.55)	29 (33.72)	0.982
Reperfusion therapy	21 (23.86)	25 (29.07)	0.528
Local flap therapy	2 (2.27)	0 (0)	0.498

BMI, body mass index; MFS, Maryland foot function score; PAD, peripheral arterial disease; NCV, nerve conduction velocity; ABI, ankle–brachial index; CKD, chronic kidney disease.

**Table 2:** comparison of surgical conditions and therapeutic outcomes.

Variable	TTT (*n* = 88)	ALTPF (*n* = 86)	*p*-value
Operative details			
Operation time (mins)	59.31 ± 18.35	274.26 ± 43.94	<0.05
Blood loss (mL)	12.11 ± 4.66	355.76 ± 144.22	<0.05
Blood transfusion [*n* (%)]	3 (3.41)	37 (43.02)	<0.05
Lower limb condition			
Wound Healing [*n* (%)]	86 (97.73)	76 (88.37)	<0.05
Ulcer recurrence [*n* (%)]	2 (2.27)	9 (10.47)	<0.05
Major amputation [*n* (%)]	1 (1.14)	6 (6.98)	<0.05
Three months postoperatively			
ABI	0.96 ± 0.17	0.84 ± 0.20	<0.05
SWMT negative^a^	82 (93.18)	67 (77.91)	<0.01
NCV (m/s)	51.30 ± 14.58	28.57 ± 16.16	<0.05
MFS	85.24 ± 7.02	79.95 ± 6.85	<0.05

^a^ SWMT, Semmes–Weinstein monofilament test; a negative result indicates intact protective sensation, defined as feeling ≥7 out of 10 sites (or ≥4 out of 4 sites in the simplified test), or perceiving pressure at key sites such as the great toe or the first metatarsal head.

After at least one year of follow-up, the ulcer healing rate was significantly higher in the TTT group than in the ALTPF group [97.73% (86/88) vs. 88.37% (76/86), *p* < 0.05]. In the TTT group, two ulcers failed to heal—one due to acute popliteal artery occlusion two weeks postoperatively and the other due to poorly controlled blood glucose with wide fluctuations, both resulting in delayed healing. In the ALTPF group, ten cases failed to heal, including four with complete flap necrosis and six with partial necrosis.

Furthermore, the ulcer recurrence rate was significantly lower in the TTT group than in the ALTPF group [2.27% (2/88) vs. 10.47% (9/86), *p* < 0.05]. Among the two recurrent cases in the TTT group, one patient underwent major amputation due to persistent heavy smoking against medical advice, whereas the other achieved ulcer healing after active wound care. In the ALTPF group, nine patients experienced recurrent ulceration after flap healing; all achieved gradual healing following glycemic control and wound drainage management. The incidence of major amputation was lower in the TTT group than in the ALTPF group [1.14% (1/88) vs. 7% (6/86), *p* < 0.05]. One patient in the TTT group underwent major amputation after recurrence, whereas six patients with flap necrosis in the ALTPF group required major amputation.

**Figure 4:**
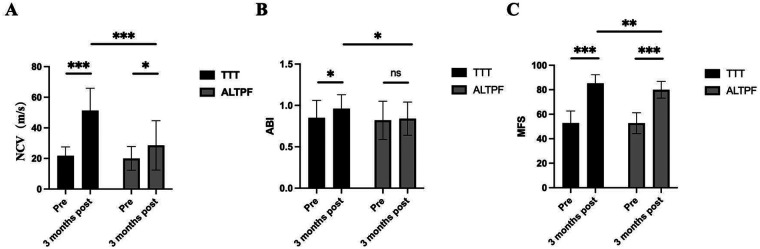
Nerve conduction velocity (NCV), ankle-brachial index (ABI), and Maryland Foot Function Score (MFS) before surgery and three months after surgery in both groups of patients. (A) NCV; (B)ABI; (C) MFS. Data are presented as mean ± SD. *P < 0.05, **P < 0.01, ***P < 0.001; ns, not significant.

As showed in Figure 4, at the three-month postoperative evaluation, the ABI results indicated that lower-limb perfusion was significantly higher in the TTT group compared with the ALTPF group (0.96 ± 0.17 vs. 0.84 ± 0.20, *p* < 0.05). Regarding neural recovery, the positive rate of the SWMT was markedly higher in the TTT group than in the ALTPF group [93.18% (82/88) vs. 77.91% (67/86), *p* < 0.05]. In addition, NCV testing demonstrated that postoperative neural responsiveness of the foot skin was significantly better in the TTT group (51.30 ± 14.58 m/s vs. 28.57 ± 16.16 m/s, *p* < 0.05). The MFS was also significantly higher in the TTT group than in the ALTPF group (85.24 ± 7.02 vs. 79.95 ± 6.85, *p* < 0.05), indicating better postoperative functional recovery of the foot.

In terms of complications, two cases of pin-tract infection occurred in the TTT group [2.27% (2/88)], both of which resolved after local wound care. In the ALTPF group, four cases of complete flap necrosis [4.65% (4/86)] and six cases of partial flap necrosis [6.98% (6/86)] were observed.

### Cases report

#### Case 1

**Figure 5:**
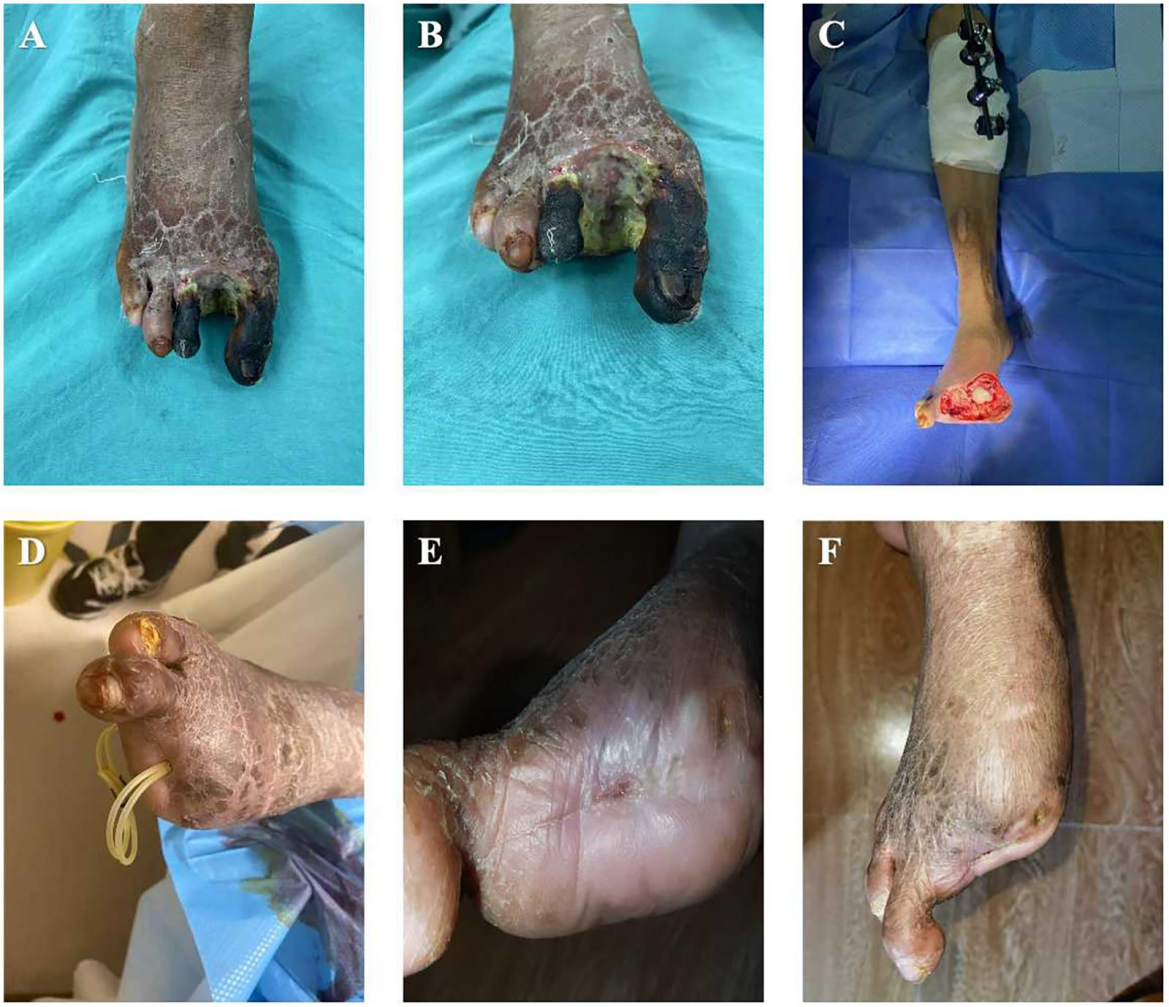
Clinical outcome of TTT treatment in a 58-year-old male patient with severe DFU. **(A,B)** Gangrene of the first to third toes on the left foot with positive bacterial culture from the wound; **(C)** After medical stabilization, TTT was performed on the left tibia with thorough wound debridement, exposing the articular surface; **(D)** At 2.5 months postoperatively, the wound was nearly healed, with only a small residual area around the drainage site; **(E,F)** At 4 months postoperatively, after removal of the drainage tube, the wound was completely healed with evident epithelialization.

A 58-year-old male patient with a 20-year history of type 2 diabetes mellitus and poor glycemic control was admitted with a non-healing ulcer on the left foot that had persisted for more than 20 weeks. Physical examination revealed necrosis of the skin over the first to third toes and the dorsum of the left foot, accompanied by foul-smelling discharge and a wound extending to the fascial layer (Figures 5A, B). TTT was performed at the proximal segment of the left tibia, with thorough debridement of all necrotic tissue until a fresh wound bed was achieved (Figure 5C).

At approximately 2.5 months postoperatively, the wound demonstrated robust granulation tissue growth and markedly accelerated healing, with a drainage tube retained to facilitate adequate drainage of secondary necrotic tissue (Figure 5D). By four months after surgery, the wound had completely healed with satisfactory skin coverage, and the foot showed an excellent appearance and functional recovery (Figures 5E, F).

#### Case 2

**Figure 6:**
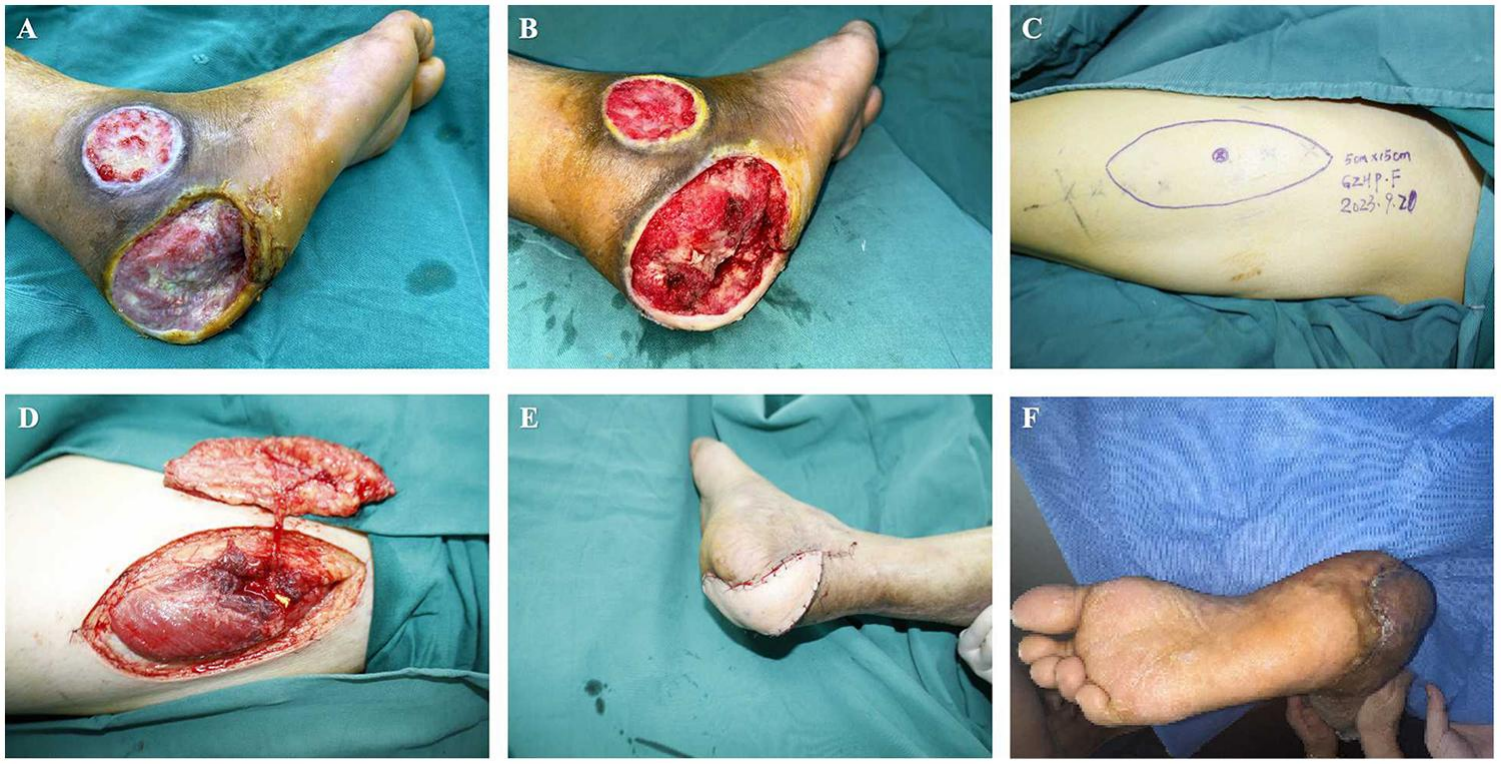
Clinical application of anterolateral thigh perforator flap (ALTPF) in a 46-year-old male patient with diabetic foot ulcer (DFU). **(A)** A large circular ulcer was present on the right heel and lateral malleolus; **(B)** The wound was debrided until fresh, reddish granulation tissue appeared; **(C)** Design of the anterolateral thigh perforator flap on the skin surface; **(D)** The flap was completely elevated, remaining connected to the donor site only by the vascular pedicle; **(E)** The flap fully covered the wound defect; **(F)** At 6 months postoperatively, the wound had not recurred, and the flap showed satisfactory survival.

A 46-year-old male patient with an 18-year history of type 2 diabetes mellitus and poor glycemic control was admitted with a chronic, non-healing ulcer of the right heel. Prior to admission, he had undergone multiple sessions of debridement, dressing changes, and anti-infective therapy, with unsatisfactory results. Physical examination revealed extensive necrosis of the skin and soft tissue of the right heel, with bone exposure and purulent discharge (Figure 6A). Intraoperatively, thorough debridement was performed to expose a fresh wound bed, followed by reconstruction of the foot defect using an ALTPF.

Postoperatively, the flap demonstrated good perfusion and satisfactory viability, and the donor site was closed primarily (Figures 6B–E). At the six-month follow-up, the flap coverage remained intact without ulcer recurrence or related complications (Figure 6F).

## Discussion

Our findings suggest that although both TTT and ALTPF are effective approaches for the treatment of severe DFU, patients treated with TTT achieved higher ulcer healing rates, along with lower rates of amputation and recurrence, effectively reducing the risk of limb loss in severe DFU. Moreover, the technical simplicity of the procedure and its lower complication rate make TTT a more practical and accessible option for clinical surgeons.

Both TTT and ALTPF transplantation have been reported in recent years as highly effective treatments for severe DFU. However, no studies to date have directly compared the therapeutic outcomes of these two approaches. Moreover, most existing research has focused primarily on ulcer healing rate and overall treatment efficacy. In contrast, the present study further investigated surgical trauma, postoperative functional recovery of the foot, and sensory restoration at the ulcer site.

In 2022, our team reported a multicenter prospective study involving seven medical centers and 1,072 patients with refractory DFU who underwent TTT. During follow-up, 1,019 patients (94.9%) achieved complete healing, with a mean healing time of 12.4 ± 5.6 weeks. Only 53 patients (4.9%) required amputation, and 33 (3.1%) experienced recurrence. Two years postoperatively, patients' health-related quality of life (HRQoL) showed significant improvement, and cortical window closure failure occurred in only eight patients (0.7%) (13).

Several clinical and experimental studies have demonstrated the efficacy of the TTT technique in promoting wound healing and limb salvage in patients with DFU.

Junpeng Liu et al. performed TTT in 98 patients with Wagner grade II or higher DFUs and achieved a remarkable healing rate of 95.83%. At three months postoperatively, both the ABI and the visual analogue scale (VAS) scores improved significantly, and no ulcer recurrence was observed during follow-up (23).

Similarly, Shuanji Ou et al. reported that among 19 patients with Wagner grade IV DFU treated with TTT, both the one-year wound healing and limb salvage rates reached 94.74%. Digital subtraction angiography (DSA) revealed thickened arteries and a dense vascular network in the lower leg and foot. Furthermore, the postoperative serum levels of angiogenic and growth factors, including VEGF, bFGF, PDGF, and EGF, were significantly elevated compared with preoperative levels (14).

In preclinical research, Yongkang Yang et al. established a TTT rat model and evaluated wound healing using laser speckle perfusion imaging, vascular perfusion, histological, and immunohistochemical analyses. Their findings demonstrated that TTT accelerated wound closure, improved the quality of regenerated skin tissue, enhanced collagen deposition, and promoted angiogenesis and immune modulation. An increased number of M2 macrophages was also observed in the wound area of the TTT group (24).

Consistent with these results, multiple experimental studies have confirmed that TTT facilitates angiogenesis, immune regulation, and inflammatory resolution, thereby creating a microenvironment conducive to tissue repair and regeneration (25, 26).

Vascular reconstruction combined with free flap transplantation has long been considered an effective limb-salvage strategy for the treatment of severe DFU. Caren Randon and colleagues (1992–2006) performed arterial reconstruction combined with free flap transplantation in 55 patients with severe DFU who were initially indicated for below-knee amputation. The 1-year and 3-year limb salvage rates were 75.8% and 64.3%, respectively, while the 1-year and 3-year amputation-free survival rates were 69.5% and 55.8%. Only one patient died during hospitalization, and 38 patients regained independent ambulation, demonstrating favorable long-term outcomes of this combined approach. The authors recommended that this strategy should be prioritized before proceeding to amputation (27).

Tae Suk Oh and colleagues (2012) conducted a logistic regression analysis of 121 patients with DFU who underwent free flap transplantation, including 90 cases reconstructed with ALTPF. The overall limb salvage rate was 84.9%, and the 5-year survival rate reached 86.8%, indicating that free flap reconstruction is a highly successful therapeutic option for DFU and significantly improves long-term patient survival (28).

Hong JP et al. further confirmed the efficacy of ALTPF reconstruction in the management of complex DFU, achieving consistently satisfactory outcomes (29–31).

The ALTPF is supplied by the descending branch of the lateral circumflex femoral artery, which provides a reliable vascular course, rich branching pattern, and high flap survival rate (32–34). The flap can be designed in single- or multilobed forms and can include portions of muscle or fascia lata to accommodate various reconstructive needs. A well-designed and appropriately harvested ALTPF not only provides effective coverage of the wound but also enhances perfusion of the surrounding tissues, thereby promoting the healing of DFU (35–38).

In the present study, the ALTPF group achieved a wound healing rate of 88% (76/86) and a major amputation rate of 7% (6/86), indicating generally satisfactory therapeutic outcomes. However, compared with the TTT group, the ALTPF group demonstrated inferior performance in terms of operative time, intraoperative blood loss, ulcer healing rate, ulcer recurrence rate, as well as postoperative functional and sensory recovery. These findings suggest that as a novel, minimally invasive technique, TTT offers significant advantages in the reconstruction of DFU.

According to our previous studies and related reports, the most common complications associated with TTT include tibial fracture at the corticotomy site, osteomyelitis, and pin-tract infection (12–14). However, the incidence of these complications remains low, and with recent advances in surgical technique and improvements in postoperative care, their occurrence has markedly decreased. In our cohort, only two cases of pin-tract infection were observed, with no instances of tibial fracture or osteomyelitis.

In contrast, the ALTPF group exhibited significantly longer operative time, greater intraoperative blood loss, and higher transfusion rates compared with the TTT group. The surgical trauma during flap harvest was substantially greater, with the extent of tissue injury in free flap transplantation approaching that of organ transplantation. Consequently, flap necrosis can have devastating physical and psychological effects on patients. Therefore, although ALTPF reconstruction can substantially improve limb salvage rates, careful evaluation and patient selection are warranted when applying this technique in individuals with poor general condition.

In this study, foot functional recovery was evaluated using the SWMT, NCV, and MFS. Chronic hyperglycemia in patients with diabetes leads to metabolic disturbances and microvascular injury, resulting in peripheral neuropathy characterized by diminished foot sensation. The SWMT and NCV are two complementary methods for assessing peripheral nerve function in diabetic foot. The SWMT is used to detect loss of protective sensation, whereas the NCV provides a quantitative assessment of nerve conduction function. The combined use of these two tests facilitates early detection, grading, and therapeutic evaluation of peripheral nerve injury.

The results demonstrated that both SWMT and NCV outcomes were superior in the TTT group compared with the ALTPF group, suggesting that TTT can significantly improve neural function in patients with DFU. Although sensory nerve anastomosis can be performed during ALTPF surgery, its ability to improve the local ischemic and inflammatory microenvironment is limited, resulting in suboptimal sensory recovery. In contrast, TTT promotes angiogenesis and soft tissue regeneration through microfracture-induced distraction, which not only facilitates wound healing but also provides a favorable microenvironment for nerve regeneration (39).

The MFS is a pain- and function-centered assessment tool that has been widely applied to evaluate functional recovery following treatment for DFU (40). In patients with DFU, postoperative improvement in the MFS typically indicates enhanced lower-limb stability, restored walking ability, and pain relief, suggesting improvements in both structural integrity and neuromuscular coordination.

The results of this study showed that postoperative MFS scores improved significantly in both groups, indicating that both TTT and ALTPF procedures can enhance lower-limb function to some extent in patients with DFU. However, the magnitude of improvement in the TTT group was markedly greater than that in the ALTPF group, suggesting that TTT provides more pronounced benefits in pain relief, gait improvement, and overall functional recovery of the foot.

The superior functional recovery observed with TTT may be attributed to its unique biological effects. Through the mechanism of “distraction angiogenesis,” TTT markedly enhances local blood perfusion while simultaneously promoting nerve regeneration, collagen remodeling, and reactivation of ischemic limb muscles (12, 26, 39, 41, 42). In contrast, although ALTPF effectively repairs soft tissue defects and restores wound integrity, its primary role is limited to local tissue coverage, with relatively modest effects on improving microcirculation and neuromuscular function in the lower limb.

Therefore, the significant improvement in MFS scores observed in the TTT group reflects its combined advantages in both structural repair and functional reconstruction. This finding indicates that TTT not only accelerates wound healing but also contributes to long-term restoration of lower-limb mobility and overall quality of life in patients with DFU.

## Limitation

This study was retrospectively designed and may be subject to selection bias and uncontrolled confounding factors. Future prospective randomized controlled trials are warranted to ensure balanced baseline characteristics between groups, minimize bias, and provide higher-quality evidence. Although the follow-up period was relatively long, long-term efficacy and recurrence of DFU require further observation over an extended period. The sample size of this study was limited to 174 patients; thus, larger studies are needed to validate the reliability of these findings. In addition, differences in surgical procedures between TTT and ALTPF led to variations in postoperative rehabilitation and follow-up protocols, which may have influenced healing outcomes and complication rates. Individual patient factors—such as glycemic control, management of comorbidities, and surgeon experience—could also have affected treatment outcomes.

## Conclusion

Both TTT and ALTPF are effective in repairing severe DFU, significantly improving wound healing rates and reducing the incidence of major amputation. Among the two, TTT demonstrated superior therapeutic efficacy, with better recovery of foot sensation and function, reduced surgical trauma, and fewer postoperative complications. Future studies with larger sample sizes and multicenter participation are warranted to further validate the reliability and generalizability of these findings.

## Data Availability

The raw data supporting the conclusions of this article will be made available by the authors, without undue reservation.
